# Age and Saving Lives in Crisis Standards of Care: A Multicenter Cohort Study of Triage Score Prognostic Accuracy

**DOI:** 10.1097/CCE.0000000000001256

**Published:** 2025-05-12

**Authors:** Michael Hermsen, Patrick G. Lyons, Govind Persad, Alice F. Bewley, Chengsheng Mao, Kaveri Chhikara, Anoop Mayampurath, Matthew Churpek, Monica E. Peek, Yuan Luo, William F. Parker

**Affiliations:** 1 Department of Medicine, University of Wisconsin School of Medicine and Public Health, Madison, WI.; 2 Department of Medicine, Washington University School of Medicine in St. Louis, St. Louis, MO.; 3 University of Denver Sturm College of Law, Denver, CO.; 4 Northwestern University Feinberg School of Medicine, Chicago, IL.; 5 Department of Medicine, University of Chicago Pritzker School of Medicine, Chicago, IL.

**Keywords:** age factors, medical ethics, prognostic factor, Sequential Organ Failure Assessment score, triage

## Abstract

**IMPORTANCE:**

Current protocols to triage life support use scores that are biased and inaccurate.

**OBJECTIVES:**

To determine if adding age to triage protocols used in disaster scenarios improves the identification of critically ill patients likely to survive.

**DESIGN, SETTING, AND PARTICIPANTS:**

Observational cohort study from March 1, 2020, to March 1, 2022, at 22 hospitals in three networks, divided into derivation (12 hospitals) and validation cohorts (ten hospitals). Participants were critically ill adults (90% COVID-19 positive) who would have needed life support during an overwhelming case surge. Life support was defined as vasoactive medications for shock, invasive or noninvasive mechanical ventilation, or oxygen therapy with Pao_2_/Fio_2_ less than 200.

**MAIN OUTCOMES AND MEASURES:**

The primary outcome was death in the intensive care unit. We fit logistic regression models using a modified Sequential Organ Failure Assessment (SOFA) score with and without age in the derivation cohort and assessed predictive performance in the validation cohort using area under the receiver operating characteristic curves (AUCs) and compared observed and predicted mortality.

**RESULTS:**

The final analysis contained 7,660 patients with 16,711 life-support episodes. In the validation cohort, the AUC for age plus SOFA was significantly higher than the AUC for SOFA alone (0.66 vs. 0.54; *p* < 0.001). SOFA score substantially overpredicted mortality (13% predicted vs. 5% observed) for younger patients (< 40 yr) and underestimated mortality (14% predicted vs. 31% observed) for older patients (> 80 yr). In contrast, age plus SOFA had good calibration overall and across age groups. The addition of age improved but did not eliminate differences between observed and predicted mortality across racial-ethnic groups.

**CONCLUSIONS AND RELEVANCE:**

Age-inclusive triage better identifies ICU survivors than SOFA alone and is more equitable. Incorporating age into prioritization algorithms could save more lives in a crisis scenario.

**KEY POINTS**

**Question:** Would adding age to U.S. Crisis Standards of Care (CSC) life-support triage protocols, which currently ignore age and rely predominantly on the Sequential Organ Failure Assessment (SOFA) score, improve the identification of critically ill patients who survive to discharge?

**Findings:** In this multicenter study of critically ill U.S. adults who required life support, age plus SOFA score predicted short-term mortality with higher area under the receiver operating characteristic curve and better calibration among various age and racial-ethnic groups than SOFA alone.

**Meaning:** These findings suggest age should be incorporated into CSC protocols to save most lives in a crisis scenario.

The COVID-19 pandemic strained healthcare systems worldwide, requiring many to operate under “crisis standards of care” (CSC) (1–3). The National Academy of Medicine defines CSC as the policies and procedures that healthcare systems employ when disasters cause a “substantial change in the usual health care operations and … level of care it is possible to deliver …” (4). Complete CSC plans contain explicit triage scores intended to allocate life support when supply is inadequate. Current protocols, however, have substantial practical and bioethical problems. The Sequential Organ Failure Assessment (SOFA) score is the cornerstone of most protocols (5–13) but was originally designed for monitoring ICU patients with sepsis (14) rather than prognosticating critical illness survival. Perhaps unsurprisingly, it has poor performance when applied to data collected before the initiation of mechanical ventilation (13, 15). Additionally, SOFA overestimates mortality in Black patients, likely related partly to the creatinine component in the renal subscore (16–20). When used for triage, this would lead to racial bias; specifically, Black patients would be allocated less life support than is appropriate (17, 19, 21).

Age has been removed from many state CSC protocols based on guidance by the Office for Civil Rights (OCR) (22–24) despite the fact that age outperforms SOFA as a triage score for mechanical ventilation (13, 15) and is strongly associated with death in critical illness generally (25–28). To our knowledge, no studies have specifically evaluated the use of age and SOFA for comprehensive critical care life support (as opposed to strictly mechanical ventilation) triage. Many CSC plans would likely be used to allocate general critical care in this way during an ICU surge crisis: some states have CSC plans which use SOFA for allocating general critical care (7, 9, 10), and others have protocols which use SOFA specifically for ventilators, but explicitly or implicitly note that such a system could be used for any “scarce resource,” including “intensive”/“critical” care (8, 11).

This multicenter observational study aimed to quantify the prognostic information gained by including age as a variable in ICU mortality predictions in a broad population of critically ill adults representative of a pandemic surge who required life support. We developed a novel SOFA + age score and compared its ability to predict short-term survival to SOFA. Finally, we aimed to determine whether the addition of age mitigates the SOFA score’s racial bias, as Black patients tend to develop critical illness at a younger age (29).

## METHODS

We conducted an observational cohort study at 22 hospitals across three health systems in the midwestern United States. This included 11 hospitals within Northwestern Medicine (NM), one hospital within UChicago Medicine (UCM), and ten hospitals within BJC HealthCare (BJC; **Supplemental Material**, https://links.lww.com/CCX/B506). UCM and NM are large academic nonprofit health systems serving the Chicagoland area. BJC is a large academic nonprofit health system in St. Louis, Missouri and surrounding areas. Some hospitals expanded ICU beds and services during the initial pandemic waves, but none activated CSC protocols or exhausted ventilator supply. The study population was adults (18 years old or older) who underwent life-support treatment for critical illness between March 1, 2020, and March 1, 2022, had a COVID-19 test result recorded, and were admitted to the ICU during their hospitalization (including outside hospital transfers). Life-support treatment was defined by receiving: 1) vasoactive medications for shock, 2) invasive or noninvasive mechanical ventilation, or 3) high-flow or facemask oxygen therapy for hypoxic respiratory failure with a respiratory SOFA subscore greater than 2. These criteria were chosen to select for patients who unambiguously needed life support during a crisis scenario. Exclusion criteria were missing or erroneous age. To simulate the composition of an ICU population during a severe COVID-19 surge, we created a final cohort of 90% COVID-19 positive admissions and 10% COVID-19 negative admissions with random sampling. We defined a patient as COVID-19 positive if they had a positive test result of any test type during or within 90 days before their hospital admission. This intentionally broad definition ensured that we captured patients with new infections, those whose hospitalizations may have represented acute complications of COVID-19, and those with an incidentally positive COVID-19 test who still required critical care.

The primary outcome was mortality, defined as death in the ICU or transfer to hospice at the end of a life-support episode (LSE). We defined the beginning of each LSE based on the time the patient received life-support treatment and the end of each LSE as when the patient was off life-support treatment for more than 8 consecutive hours.

We collected demographics, vitals, laboratory values, medications, and neurologic status for each patient. Race and ethnicity were recorded as “racial-ethnic group,” which included non-Hispanic White, non-Hispanic Black, Hispanic, and other (Supplemental Material, https://links.lww.com/CCX/B506). We calculated a modified version of the SOFA score at each hour of a patient’s hospital stay following standard practices for modernizing the score (15) (**Fig. S1**, https://links.lww.com/CCX/B506). Missing data values were generally imputed from the most recent nonmissing observation (Supplemental Material, https://links.lww.com/CCX/B506). SOFA components that were never measured were assigned a score of 0. Since higher SOFA scores correlate with higher mortality, assigning a SOFA score of 0 was the most conservative option for dealing with missing values. Additionally, in a real triage scenario, clinicians utilizing a score (such as SOFA) to triage patients for critical care are unlikely to “impute” missing values; they would calculate a SOFA score with the data they have available at the time. From these data, a 48-hour maximum SOFA score was calculated at each hour of a patient’s hospitalization, using the sum of each of the highest SOFA subscores from the prior 48-hour period.

A priori, we designated data from Chicagoland hospitals (UCM or NM) as the derivation cohort and data from hospitals near St. Louis (BJC) as the validation cohort. We derived each model from the derivation cohort and evaluated performance with both internal (ten-fold cross validation among patients from UCM) and external validation (in the independent validation cohort). We fit three different logistic regression models to predict mortality for each LSE. First, we used 48-hour maximum SOFA before life-support initiation as a continuous predictor. Second, we used categorized SOFA according to the New York State Ventilator Allocation Guidelines (SOFA ≤ 7, 8–11, ≥ 12), a common mapping of the SOFA score to allocation tiers across the United States. Finally, we fit a logistic regression with both age and 48-hour maximum SOFA score as continuous predictors and used this to develop the novel SOFA + age score. The SOFA + age score was defined as *m*(age–18) + SOFA where *m* is the ratio of the age coefficient to the SOFA coefficient.

In triaging patients who require critical care, clinicians would be most interested in comparing a patient’s predicted mortality to that of others presenting within a similar time frame; in other words, they would use the triage model to rank-order patients by their predicted mortality. As such, we assessed the rank ordering ability (statistical discrimination) of SOFA + age and SOFA using the area under the receiver operating characteristic (ROC) curve (AUC) with 95% CIs generated by 2000 bootstrap replicates stratified by primary outcome. We compared ROC curves within cohorts using paired DeLong tests. We also calculated area under the precision-recall curves (AUPRCs) with 95% CIs generated by 2000 bootstrap replicates. We assessed model calibration with calibration plots by age and racial-ethnic groups after recalibration to the baseline mortality of the validation cohort. We used the Hosmer-Lemeshow test to assess for differences in observed mortality and mortality predicted by SOFA + age and SOFA scores across deciles of each score. To check robustness, we performed a sensitivity analysis by using only the first LSE for each patient.

When comparing characteristics between derivation and validation cohorts, we used chi-square tests for categorical data (Fisher exact test was used for sex), *t* test for age, Mood’s median test for median 48-hour maximum SOFA score, and Wilcoxon rank-sum test for 48-hour maximum SOFA score distribution. We followed the Transparent Reporting of a multivariable prediction model for Individual Prognosis Or Diagnosis (TRIPOD) guidelines for prediction models (see Supplemental Material for TRIPOD checklist, https://links.lww.com/CCX/B506) (30). All procedures followed were in accordance with the ethical standards of the University of Chicago Institutional Review Board and the Helsinki Declaration of 1975. The study was approved and exempted from the informed consent requirement by the University of Chicago Institutional Review Board (IRB20-1823, approved December 7, 2020, with protocol title “Equity of scarce resource allocation: applying analytical models to inform decision making”). We performed all statistical analyses with R statistical programming environment, Version 4.2.2. All statistical tests were two-sided, and we chose a significance level of 0.05 a priori.

## RESULTS

### Study Population

There were 7,660 patients, 8,180 hospitalizations, and 16,711 LSEs in total. The derivation cohort comprised 3,634 patients and 5,545 LSEs; the validation cohort comprised 4,026 patients and 11,166 LSEs (**Table 1**; and **Fig. S2**, https://links.lww.com/CCX/B506). The derivation cohort had a higher proportion of male (59% vs. 51%; *p* < 0.001) and Hispanic (19% vs. 1.4%; *p* < 0.001) patients. The median 48-hour maximum SOFA scores at the beginning of the LSEs were different (five in derivation vs. six in validation; *p* < 0.001) and the 48-hour maximum SOFA score distributions were statistically different (*p* < 0.001; **Fig. S3**, https://links.lww.com/CCX/B506). SOFA subscore distributions were mostly similar between the two cohorts, except for the neurologic subscore; more patients had a maximum neurologic subscore in the validation cohort (**Fig. S4**, https://links.lww.com/CCX/B506). The derivation cohort had higher overall mortality (23% vs. 14%; *p* < 0.001).

**TABLE 1.** Patient Demographics and Baseline Statistics

Characteristic	Derivation Cohort	Validation Cohort
Total patients, *n*	3,634	4,026
Age^a^, mean (sd), yr	63 (16)	65 (15)
Sex^a^, *n* (%)		
Female	1,477 (41)	1,640 (41)
Male	2,157 (59)	2,065 (51)
Other/unknown	0 (0)	321 (8.0)
Racial-ethnic group^a^, *n* (%)		
Non-Hispanic White	1,446 (40)	2,191 (54)
Non-Hispanic Black	1,204 (33)	1,313 (33)
Hispanic	687 (19)	58 (1.4)
Other	297 (8.2)	464 (12)
Number of hospitals, *n*	12	10
Life-support episodes, *n*	5,545	11,166
48-hr maximum SOFA score^a^^,^^b^, median (interquartile range)	5 (4–8)	6 (4–7)
SOFA score distribution^a^^,^^b^, *n* (%)		
SOFA score 0–7	4,134 (75)	8,583 (77)
SOFA score 8–11	1,112 (20)	2,388 (21)
SOFA score 11–24	299 (5.4)	195 (1.7)
Ever on mechanical ventilation^a^^,^^b^, *n* (%)	2,612 (47)	6,456 (58)
Death or discharge to hospice^a^^,^^b^, *n* (%)	1,281 (23)	1,524 (14)

SOFA = Sequential Organ Failure Assessment.

a Statistically significant difference between derivation and validation data with *p* < 0.001. A *t* test was used to assess for difference in mean age. Fisher exact test was used to assess for difference in sex. Mood’s median test was used to assess for difference in median 48-hr maximum SOFA score. Wilcoxon rank-sum test was used to assess for difference in 48-hr maximum SOFA score distribution (using all values, rather than just SOFA score categories). A χ^2^ test was used to assess for differences in race/ethnic group, mechanical ventilation, and death.

b Characteristics calculated by life-support episode rather than by patient.

Data from the University of Chicago (one hospital) and Northwestern University (11 hospitals) were included in the derivation set. Data from Washington University in St. Louis (ten hospitals) were included in the validation set.

### Association of Age and SOFA Score With Life-Support Episode Mortality

**Figure S5*A*** (https://links.lww.com/CCX/B506) shows mortality by age group stratified by initial SOFA score in the derivation cohort. For those whose initial SOFA score was 0–7 or 8–11, increasing age was associated with higher mortality. Mortality generally increased with both increased initial SOFA score and increased age in the derivation cohort (**Fig. S5*B***, https://links.lww.com/CCX/B506). **Figure S6** (https://links.lww.com/CCX/B506) shows similar plots for the validation cohort.

### Rank Ordering Ability of Triage Scores

**Figure 1.**
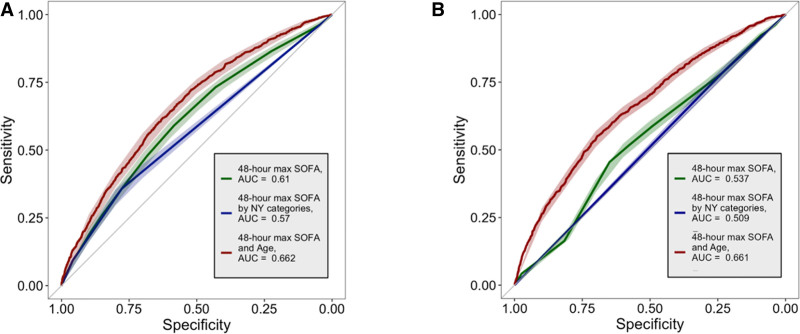
Receiver operating characteristic (ROC) curves for predictive models using Sequential Organ Failure Assessment (SOFA) score alone or SOFA score and age. ROC curves for three different logistic regression models fit to predict mortality at the end of a life-support episode (LSE) are shown. **A**, Model results from the derivation cohort. **B**, Model results from the validation cohort. Two of the models were fit using a single variable: either 48-hr maximum SOFA score at the beginning of the LSE or categories of 48-hr maximum SOFA score at the beginning of LSE based on New York (NY) State Ventilator Allocation Guidelines (SOFA 0–7, 8–11, or > 11). The last model was fit using the SOFA + age score (a novel score derived from age (yr) and 48-hr maximum SOFA score at the beginning of the LSE). The *shaded region* surrounding each curve shows the 95% CI calculated by 2000 stratified bootstrap replicates. In the derivation data, paired DeLong test for SOFA score alone vs. SOFA + age score (*p* < 0.001). Paired DeLong test for SOFA score categories vs. SOFA + age score (*p* < 0.001). In the validation data, paired DeLong test for SOFA score alone vs. SOFA + age score (*p* < 0.001). Paired DeLong test for SOFA score categories vs. SOFA + age score (*p* < 0.001). AUC = area under the receiver operating characteristic curve.

In the derivation cohort, each decade of life was associated with 1.36 times higher odds of death (OR, 1.36; 95% CI, 1.30–1.42) holding the SOFA score constant (**Table S1**, https://links.lww.com/CCX/B506). Thus, the derived SOFA + age score of *m*(age–18) + SOFA had *m* = 0.26. Each decade of life above age 18 corresponded to an independent risk of death equivalent to 2.6 SOFA score points. In the derivation cohort, SOFA + age (AUC, 0.66; 95% CI, 0.65–0.68) had better rank ordering ability than SOFA only (AUC, 0.61; 95% CI, 0.59–0.63; *p* < 0.001) and SOFA categories (AUC, 0.57; 95% CI, 0.56–0.59; *p* < 0.001) (**Fig. 1*A***; and **Tables S2–S4**, https://links.lww.com/CCX/B506). In the validation cohort, SOFA + age also performed better (AUC, 0.62; 95% CI, 0.61–0.63) when compared with SOFA only (AUC, 0.54; 95% CI, 0.52–0.55; *p* < 0.001) or SOFA categories (AUC, 0.51; 95% CI, 0.50–0.52; *p* < 0.001) (**Fig. 1*B***; and Tables S2–S4, https://links.lww.com/CCX/B506). See **Supplemental Results** of internal cross validation (https://links.lww.com/CCX/B506).

### AUPRCs of Triage Scores

In the derivation cohort, SOFA + age had the highest AUPRC (0.37; 95% CI, 0.35–0.40), compared with SOFA only (AUPRC, 0.32; 95% CI, 0.29–0.34) and SOFA categories (AUPRC, 0.28; 95% CI, 0.26–0.29). Similarly, SOFA + age had the highest AUPRC in the validation cohort (AUPRC, 0.20; 95% CI, 0.18–0.21) compared with SOFA only (AUPRC, 0.13; 95% CI, 0.12–0.14) and SOFA categories (AUPRC, 0.13; 95% CI, 0.12–0.14).

### Calibration of Triage Scores

**Figure 2.**
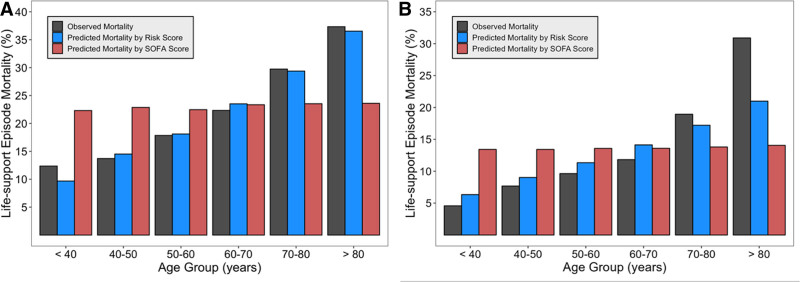
Life-support episode (LSE) mortality by age group in derivation and validation cohorts. **A**, Observed and predicted LSE mortality at various age groups for the derivation cohort. **B**, Observed and predicted LSE mortality at various age groups for the validation cohort. Risk Score is the SOFA + age score. Both the Sequential Organ Failure Assessment (SOFA) + age score and SOFA score were recalibrated to the validation data before making predictions in the validation data.

The SOFA + age score was well calibrated in the validation cohort with no significant difference in observed and predicted mortality (*p* = 0.50; **Fig. S7**, https://links.lww.com/CCX/B506). In contrast, the SOFA score was poorly calibrated (*p* < 0.001; Fig. S7, https://links.lww.com/CCX/B506). In the validation cohort, patients under 40 years (*n* = 1027) had a mean SOFA score of 6.25 corresponding to a predicted mortality of 13% by the recalibrated SOFA-only model, but only 5% died. Patients older than 80 years (*n* = 1113) had a mean SOFA score of 5.30 corresponding to a predicted mortality of 14% by the recalibrated SOFA-only model, yet 31% died (**Table S5** for all age groups in both cohorts, https://links.lww.com/CCX/B506). Additionally, mortality predicted by SOFA + age score was closer to the observed mortality for nearly all age groups in both the derivation and validation datasets (**Fig. 2**). Calibration curves for each model are shown in **Figures S8–S10** (https://links.lww.com/CCX/B506).

### Calibration of Triage Scores by Racial-Ethnic Groups

**Figure 3.**
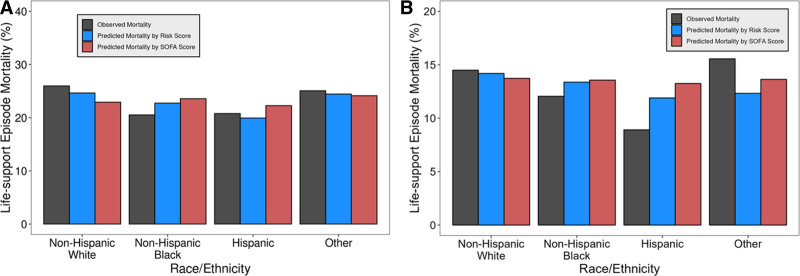
Life-support episode (LSE) mortality by racial-ethnic groups. Observed and predicted LSE mortality for different race/ethnicity groups. **A**, Data from the derivation set. **B**, Data from the validation set. SOFA = Sequential Organ Failure Assessment. Risk Score is the SOFA + age score.

In general, SOFA + age score was better calibrated to observed mortality than SOFA alone across racial-ethnic groups analyzed (**Fig. 3**). In the derivation cohort, mortality predicted by SOFA + age score was closer to the observed mortality than mortality predicted by SOFA alone for all racial-ethnic groups: non-Hispanic White individuals (25.9%, 24.6%, and 22.9% for observed, SOFA + age-predicted, and SOFA-predicted, respectively), non-Hispanic Black individuals (20.5%, 22.7%, 23.6%), Hispanic individuals (20.8%, 19.9%, 22.3%), and individuals in racial-ethnic group “Other” (25.1%, 24.4%, 24.1%; Fig. 3*A*). The same was true in the validation cohort for non-Hispanic White individuals (14.5%, 14.2%, and 13.7% for observed, SOFA + age-predicted, and SOFA-predicted, respectively), non-Hispanic Black individuals (12.0%, 13.4%, 13.6%), and Hispanic individuals (8.9%, 11.9%, 13.2%; Fig. 3*B*). Within racial-ethnic group “Other” in the validation cohort, mortality predicted by SOFA score alone was closer to observed mortality (observed 15.6%, SOFA + age-predicted 12.3%, SOFA-predicted 13.6%).

### Sensitivity Analyses

Results using only the first LSE for each encounter were similar (Supplemental Results and **Figs. S11–S14**, https://links.lww.com/CCX/B506).

## DISCUSSION

In this independently validated multicenter observational study of 7660 severely critically ill patients at 22 hospitals, we found that using age in addition to SOFA score substantially improved predictions of critical illness survival in a respiratory pandemic surge population. Each decade of life increased mortality risk by the equivalent of 2.6 SOFA score points—a similar increase to initiating invasive mechanical ventilation or vasoactives. Mortality increased with age while holding the initial SOFA score constant. We quantified the consequences of using SOFA only to predict mortality for various age groups (for younger patients, overprediction of mortality and less resource allocation; for elderly patients, underprediction of mortality and more resource allocation). We also showed that a new triage score—SOFA + age—utilizing both age and SOFA performs better than SOFA alone; such a triage score could be used to rank patients for receipt of scarce life-support resources. Finally, the SOFA + age score was better calibrated to observed mortality than SOFA alone among patients identifying as non-Hispanic White, non-Hispanic Black, and Hispanic, although disparities remained.

After the initial months of the COVID-19 pandemic, it became clear that in a pandemic crisis scenario, critical care life support as a comprehensive package—rather than a specific device like the mechanical ventilator—would become scarce. In contrast to prior studies that only focused on patients treated with mechanical ventilation, our results demonstrate that ignoring age in ICU triage will lead to more deaths in a crisis. SOFA + age had a much better AUC than either of the SOFA-only models (SOFA as linear predictor or categories) for predicting short-term mortality. The results of previously published studies on ICU mortality make it unsurprising that age adds prognostic information to the SOFA score in this context (25–27). Yet age is often omitted as a clinical criterion in CSC protocols across the country, despite its widespread use to allocate other scarce medical resources, such as COVID-19 vaccines and antivirals (31, 32).

Legal fears may explain the omission of age. In 2020, the Health and Human Services (HHS) OCR stated in a nonbinding guidance document that medical care should not be denied “on the basis of stereotypes, assessments of quality of life, or judgments about a person’s relative “worth” based on the presence or absence of disabilities or age” (33). In response, many states deemphasized or dropped age completely from their CSC via OCR’s rapid resolution process. OCR’s informal guidance was never tested or clarified in court (6, 22–24, 34, 35). However, the Age Discrimination Act of 1975 and its implementing regulations permit much broader use of age than analogous statutes concerning race, sex, and disability discrimination. OCR also states “age can be explicitly taken into account if: (a) Age is used as a measure or approximation of one or more other characteristics … [that] must be measured or approximated in order for the normal operation of the program or activity to continue … can be reasonably measured or approximated by the use of age; and … are impractical to measure directly on an individual basis” (**Fig. S15**, https://links.lww.com/CCX/B506). Short-term mortality cannot be objectively “measured” a priori and obviously must be approximated by a score. The SOFA + age model does not make quality-of-life judgments, stereotypes, or social worth assessments. Rather, like antiviral, vaccine, and deceased donor organ allocation (all HHS approved) (36), SOFA + age relies heavily on age because it is an indispensable variable for estimating short-term clinical benefit from life-support treatment.

Arguably, the OCR should be more concerned that the SOFA score is biased against Black patients (16–18). Black patients’ rates of chronic kidney disease (CKD) exceed other groups’, and the SOFA score’s use of creatinine values alone penalizes individuals with CKD similarly to those with acute renal failure (16–20). Black patients, therefore, have inaccurately high SOFA scores, inappropriately reducing their access to scarce life-saving resources in a triage scenario. Similarly, our study found that SOFA overestimated mortality in Black patients in both derivation and validation sets. The addition of age was insufficient to fully debias the SOFA score for Black individuals: while SOFA + age marginally outperformed SOFA, it still overestimated mortality. For Hispanic patients, our validation cohort was limited by small sample size. In the derivation cohort, SOFA score also overpredicted mortality among Hispanic patients. In general, the SOFA + age score was better calibrated to mortality by racial-ethnic groups than SOFA alone, although the improvements were often small. Further study is needed to refine SOFA + age model to better predict outcomes across racial-ethnic groups. Even though adding race to the score could theoretically counteract the bias conferred by the renal component of the SOFA score, for practical, sociopolitical, and legal reasons, life-support allocation scores cannot not use a patient’s race or ethnicity as a variable in the score as that would explicitly prioritize one patient over another by race. Instead, the underlying prediction model (in this case, SOFA) should be updated to eliminate the bias.

While SOFA + age outperformed the currently used SOFA score, there is still much room for improvement. Future models may benefit from considering SOFA components separately or even dispensing with SOFA altogether given its limitations discussed above; regardless, our results suggest age should be incorporated or at least considered in future models. One reason SOFA score has been used is simplicity. While newer, more sophisticated models may be able to generate more accurate mortality predictions, they are also far more complicated and none of them (to our knowledge) are currently being used or were used in the past for life-support triage. One of the criticisms of more complicated machine-learning models is that they can be difficult to interpret and function somewhat like a “black box,” an undesirable trait when used for triaging critical care for saving lives. Such models are unlikely to be adopted by public health agencies for use in this manner, at least until further research and public awareness of machine learning improve knowledge of and trust in more complicated models.

Strengths of this work include its inclusion of 2 years of data from many hospitals, including a mix of urban/suburban/rural and academic/community hospitals. The multicenter approach allowed a larger study population and external validation of SOFA + age. Our pragmatic use of readily available electronic health record data means SOFA + age could be calculated in other institutions and populations. Demographic differences between the derivation and validation cohorts (e.g., race-ethnicity, mortality) show that our results are robust to varying patient populations.

Our article has a few limitations. First, our multihospital data were neither standardized nor harmonized, leading to inevitable discrepancies in the data or coding of certain variables. Second, there were few Hispanic patients in the validation cohort (1.4%). Third, the SOFA subscore distributions were different between the two cohorts (Fig. S4, https://links.lww.com/CCX/B506); most prominently, patients in the validation cohort more frequently had maximal neurologic subscores. These differences may underlie the inconsistent relationship between SOFA and mortality between our cohorts. These findings highlight the fact that EHR-based SOFA scores are vulnerable to local case mix, data quality, and documentation practices, especially before receipt of life support, which further underscores their inappropriateness for predicting mortality in this setting. Finally, we chose final cohorts to be 90% COVID-19 positive, rather than using a general ICU population, to represent a pandemic surge population. Therefore, the SOFA + age score needs validation in other crisis scenarios.

## CONCLUSIONS

Current protocols, which would be used to triage life support in future pandemics or other public health crises, are inaccurate, biased, and not currently optimized to save the most lives. Protocols which incorporate age as a predictor of ICU survival could increase the number of lives saved (the explicit goal of most protocols) while also enabling a more equitable ranking system. Omitting age from CSC triage protocols actively biases the protocols against younger patients who are likelier to benefit from life support. In short, age substantially improves the prediction of short-term survival in critically ill adults and should be incorporated into CSC triage scores, although further research is needed to refine these scores.

## Supplementary Material


