# Supplementary material for: Age and Saving Lives in Crisis Standards of Care: A Multicenter Cohort Study of Triage Score Prognostic Accuracy
Source: Crit Care Explor. 2025 May 12;7(5):e1256. doi: 10.1097/CCE.0000000000001256 (PMC12074069; doi:10.1097/CCE.0000000000001256)
Supplement: Supplementary file 1 [file cc9-7-e1256-s001.pdf]

Supplemental Material

Article Title: Age and Saving Lives in Crisis Standards of Care: A multi-center cohort study of triage score prognostic accuracy.

Table of Contents

Section 1: Supplemental Methods

Section 2: TRIPOD Checklist

Section 3: Supplemental Results

Section 4: Supplemental Exhibits
Table S1. Logistic regression for life support episode mortality using age and SOFA score.
Table S2. Logistic regression for life support episode mortality using SOFA score.
Table S3. Logistic regression for life support episode mortality using SOFA score categories.
Table S4. Logistic regression for life support episode mortality using SOFA+Age score.
Table S5. Average SOFA scores, Predicted Mortality, and Observed Mortality by Age Group.
Figure S1. Rules for calculating SOFA score.
Figure S2. Flow diagram for study encounters.
Figure S3. SOFA Score Distribution in Derivation and Validation Cohorts.
Figure S4. SOFA Sub-score Distributions in Derivation and Validation Cohorts.
Figure S5. Association between Mortality and Age in the Derivation Cohort.
Figure S6. Association between Mortality and Age in the Validation Cohort.
Figure S7. Life Support Episode Mortality by Deciles of SOFA+Age Score.
Figure S8. Calibration Curves for Logistic Regression using SOFA Score in Derivation and Validation Cohorts.
Figure S9. Calibration Curves for Logistic Regression using SOFA Categories in Derivation and Validation Cohorts.
Figure S10. Calibration Curve for Logistic Regression using SOFA Score and Age in Derivation and Validation Cohorts.
Figure S11. Receiver-operator characteristic curves for predictive models using SOFA score or SOFA+Age score (first LSE only).
Figure S12. Life Support Episode Mortality by Deciles of SOFA+Age or SOFA Score (first LSE only).
Figure S13. Life support episode mortality by age group in derivation and validation sets (first LSE only).
Figure S14. Life support episode mortality by racial-ethnic groups (first LSE only).
Figure S15. HHS OCR Statement on Civil Rights and COVID-19.

For access to R scripts and further explanation of coding details, see https://github.com/08wparker/age_life_support_triage.

Section 1: Supplemental Methods

As described in the main text, we *a priori* designated 12 hospitals within the Northwestern Medicine and UChicago Medicine healthcare systems as the derivation cohort and 10 hospitals within the BJC HealthCare system as the validation cohort.

Northwestern Medicine is a nonprofit academic health system in the Chicago area. Northwestern Memorial Hospital is a 900-bed quaternary referral center and the primary teaching hospital of the Northwestern University Feinberg School of Medicine. There are 11 hospitals within this network, and all were included in this study: Northwestern Memorial Hospital, Central DuPage Hospital, Delnor Hospital, Lake Forest Hospital, Kishwaukee Hospital, Valley West Hospital, Marianjoy Rehabilitation Hospital, Huntley Hospital, McHenry Hospital, Woodstock Hospital, and Palos Hospital.

UChicago Medicine is a nonprofit academic health system in the Chicago area. The main Hyde Park campus includes both the Center for Care and Discovery and Mitchell Hospital, together a 500-bed quaternary referral center and the primary teaching location of the University of Chicago Pritzker School of Medicine. Only data from the main campus was included, as we did not have access to data from the smaller affiliate hospitals within UCM.

BJC is a nonprofit health academic system comprising 14 hospitals in the St. Louis, MO metropolitan area, mid-Missouri, and southern Illinois. Barnes Jewish Hospital, BJC's urban academic hospital is a 1,300-bed quaternary referral center and the primary teaching hospital of the Washington University School of Medicine. Of these 14 hospitals, we excluded St. Louis Children's Hospital, The Rehabilitation Institute of St. Louis (not an acute care hospital), and 2 hospitals which were not part of BJC for the entirety of the study period. Thus, the included hospitals were Barnes-Jewish Hospital, Christian Hospital, Missouri Baptist Medical Center, Alton Memorial Hospital, Barnes-Jewish St. Peters Hospital, Barnes-Jewish West County Hospital, Progress West Hospital, Parkland Health Center, Missouri Baptist Sullivan Hospital, and Memorial Hospital.

For this study, race and ethnicity were recorded as a single variable, "racial-ethnic group", which included Non-Hispanic White, Non-Hispanic Black, Hispanic, and "Other". Patients of any race whose ethnicity was recorded as Hispanic were placed in the Hispanic racial-ethnic group. The "Other" group included Asian, Chinese, Korean, Vietnamese, Filipino, Asian Indian, Asian/Mideast Indian, Other Asian, American Indian or Alaska Native, Native Hawaiian or Other Pacific Islander, Native Hawaiian, Other Pacific Islander, and individuals with no race specified. These were combined due to small numbers of individuals in these groups and to standardize across institutions.

Life-support treatment was defined by receiving (1) vasoactive medications for shock, (2) invasive or non-invasive mechanical ventilation, or (3) high-flow or facemask oxygen therapy for hypoxic respiratory failure with a respiratory SOFA sub-score > 2 (e.g., PaO_2/FiO_2 ratio < 200). We defined the beginning of each LSE based on the time the patient received life-support treatment and the end of each LSE as when the patient was off life-support treatment for more

than 8 consecutive hours. An LSE could begin in the emergency department (ED) or general wards, but patients had to be transferred to the ICU to be included (patients who died in the ED or general wards prior to transfer were excluded). Patients could have had multiple LSEs during each hospital admission.

We calculated a modified version of the SOFA score at each hour of a patient's hospital stay following standard practices for modernizing the score[12,26]: (1)cardiovascular sub-scores were based on the number of vasopressors (including norepinephrine, epinephrine, phenylephrine, vasopressin, angiotensin, and dopamine), rather than pressor type or dose, (2) respiratory sub-scores preferentially used PaO2/FiO2 (P/F) ratios, but used SaO2/FiO2 (S/F) ratios when P/F ratios weren't available or the P/F ratio was more than 4 hours old (e.g., the last arterial blood gas was more than 4 hours ago), and (3) renal sub-scores used only creatinine and not urine output (see **e-Table 1**for details).

Missing data values were imputed from the most recent non-missing observation, with two exceptions: dialysis (renal sub-score of 4) was carried forward for a maximum of 72 hours, and blood gas PaO2 values were carried forward for a maximum of 4 hours.

SOFA sub-scores which were never measured were assigned a score of 0. Since higher SOFA scores correlate with higher mortality, assigning a SOFA sub-score of 0 was the most conservative option for dealing with missing components. This method also makes sense for a variety of practical reasons: 1) SOFA components which are missing may be missing due to patients' clinical status. For example, the clinician may not order a bilirubin level if they suspect it is normal; in such cases, the best estimate for a Liver sub-score would be 0. 2) In a real triage scenario, clinicians utilizing a score (such as SOFA) to triage patients for critical care are unlikely to "impute" missing values; they would calculate a SOFA score with the data they have available at the time.

Prior to model derivation, we did not rebalance classes (of note, 23% of LSEs resulted in the outcome of death) since this would not alter the magnitude of the effect size for the dependent variables in logistic regression.

Section 2: TRIPOD Statement Checklist

Topic	Item	Page(s)	Comments
Title	1	1	
Abstract	2	2-3	
Background and objectives	3a	4-5	
	3b	5	
Source of data	4a	5-6	
	4b	5	
Participants	5a	5	
	5b	5-6	
	5c	N/A	Not evaluating an intervention or treatment.
Outcome	6a	6	
	6b	N/A	Outcome was not blinded to researchers.
Predictors	7a	6-7	
	7b	N/A	Outcome was not blinded to researchers.
Sample size	8	5-6	Retrospective, determined by inclusion/exclusion criteria
Missing data	9	6-7	
Statistical analysis methods	10a	7	
	10b	7	
	10c	7	
	10d	7-8	
	10e	N/A	Model not updated based on validation data.
Risk Groups	11	8	
Development vs. validation	12	5, 7	
Participants	13a	5-6, 8-9, Figure S2	
	13b	8-9, Table 1	
	13c	8-9, Table 1	
Model development	14a	8-9, Table 1	
	14b	9, Tables S1-S4	
Model specification	15a	9	
	15b	7, 9	
Model performance	16	9-10	
Model updating	17	N/A	Model not updated based on validation data.
Limitations	18	15-16	
Interpretation	19a	8-11, 14-16	
	19b	12-15	
Implications	20	12-16	
Supplementary information	21	5	Supplementary Material available online.
Funding	22	1	

Section 3: Supplemental Results

In the 10-fold cross validation of patients from UCM, the AUC for SOFA and age was 0.68 (95% CI 0.65 – 0.71). The AUC for the SOFA score was 0.65 (95% CI 0.62 – 68; $p = 0.005$ by paired DeLong's test when compared to SOFA and age). Model coefficients were similar to those reported in the full derivation cohort (age: 0.028, 95% CI 0.020 – 0.036; SOFA: 0.162 (95% CI 0.123 – 0.201), intercept: -2.401 (95% CI -2.683 – -2.119); and SOFA-only: 0.164 (95% CI 0.127 – 0.201), intercept: -4.181 (95% CI -4.786 – -3.576)).

When restricting the analysis to only the first LSE for each encounter, we obtained the following results. In the derivation cohort, there were 3,763 LSEs with 985 ending in death (26%) and 2,146 requiring mechanical ventilation at some point (57%). The median 48-hour maximum SOFA score at the beginning of the LSE was 5 (IQR 4 – 7). There were 2,909 (77%) LSEs with SOFA score 0 – 7, 705 (19%) with SOFA score 8 – 11, and 149 (4.0%) with SOFA score greater than 11. In the validation cohort, there were 4,417 LSEs with 966 ending in death (22%) and 2,079 requiring mechanical ventilation (47%). The median 48-hour maximum SOFA score was 4 (IQR 3 – 7). There were 3,888 (88%) LSEs with SOFA score 0 – 7, 508 (12%) with SOFA score 8 – 11, and 21 (0.5%) with SOFA score greater than 11. Patient demographics in both derivation and validation cohorts remained the same as those reported in the main manuscript.

In the validation cohort, the AUC for SOFA+Age was 0.64 (95% CI 0.62 – 0.65). The AUCs for the models using SOFA score and SOFA categories only were 0.55 (95% CI 0.53 – 0.57) and 0.52 (95% CI 0.51 – 0.53). The model using SOFA+Age score showed better ability to predict LSE mortality than either of the other models ($p < 0.001$ by paired DeLong's test). **Figure S11** shows the receiver-operator characteristic curves for the three models.

SOFA+Age showed good calibration with no significant difference in observed mortality across deciles of the SOFA+Age score ($p = 0.2$ by Hosmer-Lemeshow, see **Figure S12**). In contrast, there were significant differences in observed mortality across deciles of the SOFA score alone ($p < 0.001$ by Hosmer-Lemeshow, see **Figure S12**). The SOFA+Age score predicted LSE mortality more accurately than SOFA alone across every age group (see **Figure S13**) and most race/ethnicity groups (see **Figure S14**).

Section 4: Supplemental Tables and Figures

Table S1. Logistic regression for life support episode mortality using age and SOFA score.

	Value
**Number of outcomes**	1281
**Number of non-outcomes**	4264
**Intercept**	-3.92
**95% CI**	(-4.26, -3.59)
**Coefficient: Age (years)**	0.031
**95% CI**	(0.026, 0.035)
**Odds Ratio**	1.031
**Odds Ratio 95% CI**	(1.026, 1.036)
**Coefficient: SOFA score**	0.12
**95% CI**	(0.098, 0.014)
**Odds Ratio**	1.13
**Odds Ratio 95% CI**	(1.10, 1.15)
**AIC**	5665.8

Table S1 shows results from the logistic regression model trained on the derivation cohort to predict mortality at the end of the LSE. This model was not run on the validation cohort, so only derivation data is shown. Predictors were age (in years) and 48-hour maximum SOFA score at the beginning of the LSE. A 1-year increase in age was associated with 1.03 higher odds of death; similarly, a 10-year increase in age was associated with 1.36 higher odds of death (using a coefficient for age in decades of 0.31). A 1-point increase in SOFA score was associated with 1.13 higher odds of death. To calculate the value of m used in our novel risk score, we took the ratio of the age coefficient to SOFA score coefficient (m = 0.26). CI = confidence interval, AIC = Akaike information criterion

Table S2. Logistic regression for life support episode mortality using SOFA score.

	Derivation Cohort	Validation Cohort
**Number of outcomes**	1281	1524
**Number of non-outcomes**	4264	9642
**Intercept**	-1.95	-1.50
**95% CI**	(-2.10, -1.81)	(-1.65, -1.35)
**Coefficient: SOFA score**	0.12	-0.06
**95% CI**	(0.10, 0.14)	(-0.08, -0.03)
**Odds Ratio**	1.13	0.94
**Odds Ratio 95% CI**	(1.11, 1.15)	(0.92, 0.96)
**AIC**	5858.2	8880

Table S2 shows results from the logistic regression model to predict mortality at the end of the LSE using SOFA score as a predictor. Results are shown for data from both the derivation cohort and validation cohort. The predictor was 48-hour maximum SOFA score at the beginning of the LSE. CI = confidence interval, AIC = Akaike information criterion

Table S3. Logistic regression for life support episode mortality using SOFA score categories.

	Derivation Cohort	Validation Cohort
**Number of outcomes**	1281	1524
**Number of non-outcomes**	4264	9642
**Intercept: SOFA score < 8**	-1.39	-1.82
**95% CI**	(-1.47, -1.32)	(-1.88, -1.76)
**Coefficient: SOFA score 8 - 11**	0.57	-0.10
**95% CI**	(0.42, 0.72)	(-0.24, 0.03)
**Odds Ratio**	1.77	0.90
**Odds Ratio 95% CI**	(1.52, 2.05)	(0.79, 1.03)
**Coefficient: SOFA score > 11**	0.99	-0.10
**95% CI**	(0.75, 1.24)	(-0.54, 0.31)
**Odds Ratio**	2.70	0.91
**Odds Ratio 95% CI**	(2.11, 3.44)	(0.58, 1.36)
**AIC**	5900.4	8903.6

Table S3 shows results from the logistic regression model to predict mortality at the end of the LSE using SOFA score categories as predictors. Results are shown for data from both the derivation cohort and validation cohort. The predicters were SOFA score < 8, SOFA score 8 – 11, and SOFA score > 11. This categorization was based on the New York State Ventilator Allocation Guideline groups. CI = confidence interval, AIC = Akaike information criterion

Table S4. Logistic regression for life support episode mortality using SOFA+Age score.

	Derivation Cohort	Validation Cohort
**Number of outcomes**	1281	1524
**Number of non-outcomes**	4264	9642
**Intercept**	-3.37	-3.80
**95% CI**	(-3.64, -3.11)	(-4.07, -3.53)
**Coefficient: SOFA+Age score**	0.12	0.11
**95% CI**	(0.10, 0.13)	(0.09, 0.12)
**Odds Ratio**	1.13	1.11
**Odds Ratio 95% CI**	(1.11, 1.14)	(1.10, 1.13)
**AIC**	5664.4	8659

Table S4 shows results from the logistic regression model to predict mortality at the end of the LSE using SOFA+Age score as a predictor. Results are shown for data from both the derivation cohort and validation cohort. The predicter was the SOFA+Age score, a novel risk score utilizing both age and SOFA score. The SOFA+Age score was calculated by m (age – 18) + SOFA. To calculate the value of m, we took the ratio of the age coefficient to SOFA score coefficient (m = 0.26) in the age plus SOFA score model trained on the derivation data. CI = confidence interval, AIC = Akaike information criterion

Table S5. Average SOFA scores, Predicted Mortality, and Observed Mortality by Age Group.

Derivation Cohort						
**Age Group (years)**	**< 40**	**40 – 50**	**50 – 60**	**60 – 70**	**70 – 80**	**> 80**
**Total LSEs**	550	583	1053	1482	1143	734
**Mean SOFA Score**	5.48	5.70	5.56	5.94	6.10	6.16
**Predicted Mortality**	22.3%	22.9%	22.5%	23.4%	23.5%	23.6%
**Observed Mortality**	12.4%	13.7%	17.9%	22.3%	29.7%	37.3%
**Validation Cohort**						
**Age Group (years)**	**< 40**	**40 – 50**	**50 – 60**	**60 – 70**	**70 – 80**	**> 80**
**Total LSEs**	1027	1107	2285	3358	2276	1113
**Mean SOFA Score**	6.25	6.26	6.02	5.99	5.68	5.30
**Predicted Mortality**	13.4%	13.4%	13.6%	13.6%	13.8%	14.1%
**Observed Mortality**	4.6%	7.7%	9.6%	11.8%	18.9%	30.9%

Table S5 shows the total number of LSEs, mean 48-hour maximum SOFA score at the beginning of each LSE, mortality predicted by SOFA score alone, and observed mortality for various age groups in both the derivation cohort (top) and validation cohort (bottom). LSE = life support episode

\

Figure S1. Rules for calculating SOFA score.

Category	Sub-score				
	0	1	2	3	4
Cardiovascular	MAP > 70 mmHg and no pressors	MAP < 70 mmHg and no pressors	Dobutamine alone	1 pressor	2 or more pressors
Respiratory	P/F ≥ 400	300 ≤ P/F < 400	a) 200 ≤ P/F < 300 or b) P/F < 200 and not receiving ventilatory support	100 ≤ P/F < 200 and receiving ventilatory support	P/F < 100 and receiving ventilatory support
	S/F ≥ 400	315 ≤ S/F < 400	a) 235 ≤ S/F < 315 or b) S/F < 235 and not receiving ventilatory support	150 ≤ S/F < 235 and receiving ventilatory support	S/F < 150 and receiving ventilatory support
Renal	Cr < 1.2	1.2 ≤ Cr < 2.0	2.0 ≤ Cr < 3.5	3.5 ≤ Cr < 5.0	Cr ≥ 5.0 or on dialysis
Liver	Tbil < 1.2	1.2 ≤ Tbil < 2.0	2.0 ≤ Tbil < 6.0	6.0 ≤ Tbil < 12.0	Tbil ≥ 12.0
Coagulation	PLT ≥ 150	100 ≤ PLT < 150	50 ≤ PLT < 100	20 ≤ PLT < 50	Platelets < 20
Neurologic	GCS 15	GCS 13-14	GCS 10-12	GCS 6-9	GCS 0-5

Figure S1 shows the rules used to calculate the SOFA score. Respiratory sub-scores were assigned based on the P/F ratio when available; if the P/F ratio was missing or PaO2 value was more than 4 hours old, S/F ratio was used. Ventilatory support includes high-level ICU ventilatory support such as mechanical ventilation, CPAP, BiPAP, HFNC, etc. Low-flow nasal cannula was not considered ventilatory support. Creatinine and total bilirubin values measured in mg/dL. Platelet counts measured in units of 10^3 per μL. MAP = mean arterial pressure, P/F = PaO2/FiO2 ratio, S/F = SpO2/FiO2 ratio, Cr = creatinine, Tbil = total bilirubin, PLT = platelet count, GCS = Glasgow Coma Scale.

Figure S2. Flow diagram for study encounters.

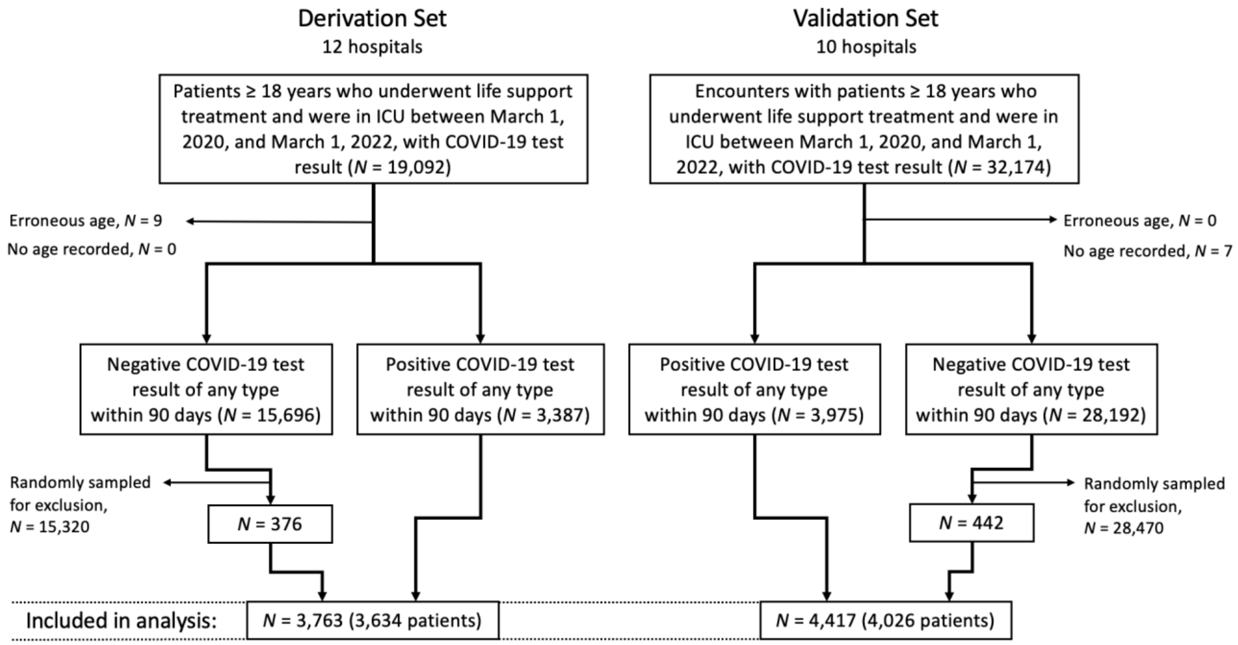

Figure S2 shows the flow diagram for encounters. Encounters were considered to have a positive COVID-19 test if they had a positive test result of any type within 90 days prior to the start of the encounter. Encounters with a negative COVID-19 test were randomly sampled without replacement so that the final derivation and validation sets would be comprised of 90% COVID-19 positive encounters and 10% COVID-19 negative encounters. Encounters from hospitals within UChicago Medicine (1 hospital) and Northwestern Medicine (11 hospitals) were included in the derivation set. Encounters from hospitals within BJC HealthCare (10 hospitals) were included in the validation set. ICU = intensive care unit

Figure S3. SOFA Score Distribution in Derivation and Validation Cohorts

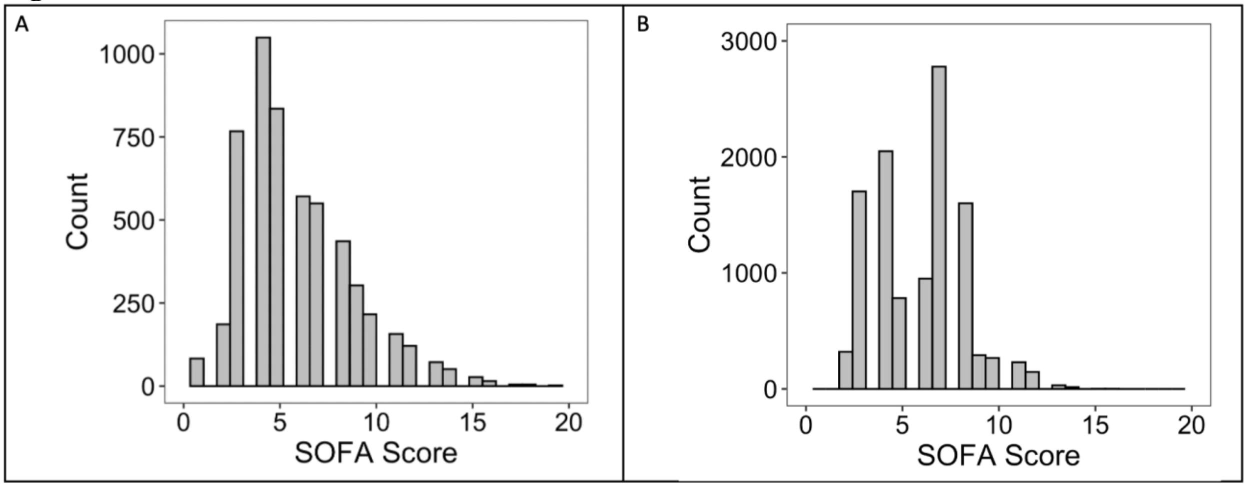

Figure S3 shows histograms for the 48-hour maximum SOFA score at the beginning of each LSE. Panel A shows data from the derivation cohort and panel B shows data from the validation cohort. The derivation and validation SOFA score distributions were different by Wilcoxon Rank Sum test ($p < 0.001$).

Figure S4. SOFA Sub-score Distributions in Derivation and Validation Cohorts.

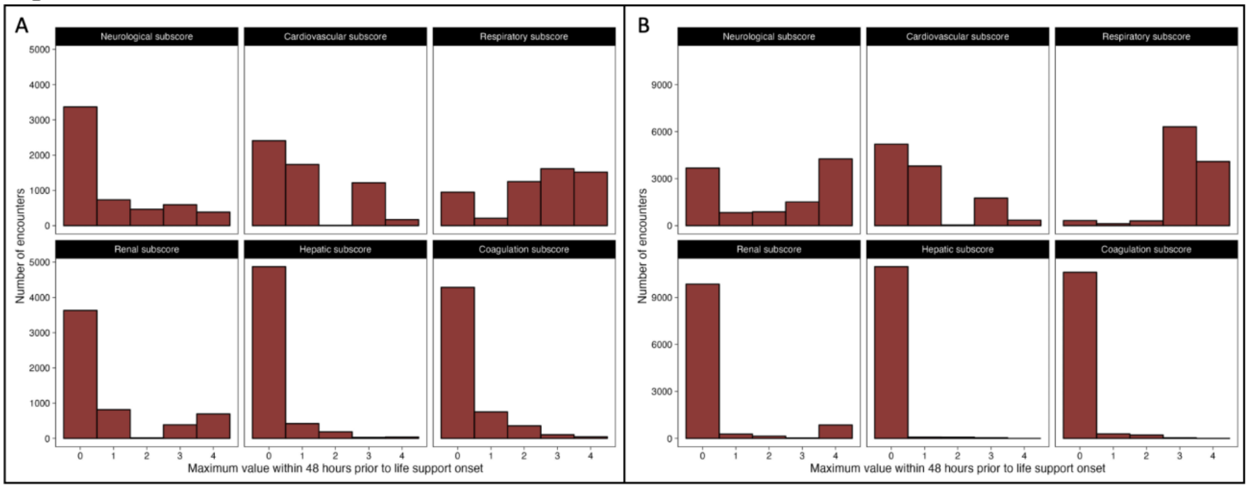

e-Figure 4, panel A shows the distributions of the SOFA sub-scores for the derivation cohort. Panel B shows the distributions of the SOFA sub-scores for the validation cohort. In both cases, values shown are 48-hour maximum value for that sub-score at the beginning of each LSE. SOFA = Sequential Organ Failure Assessment, LSE = Life Support Episode

Figure S5. Association between Mortality and Age in the Derivation Cohort.

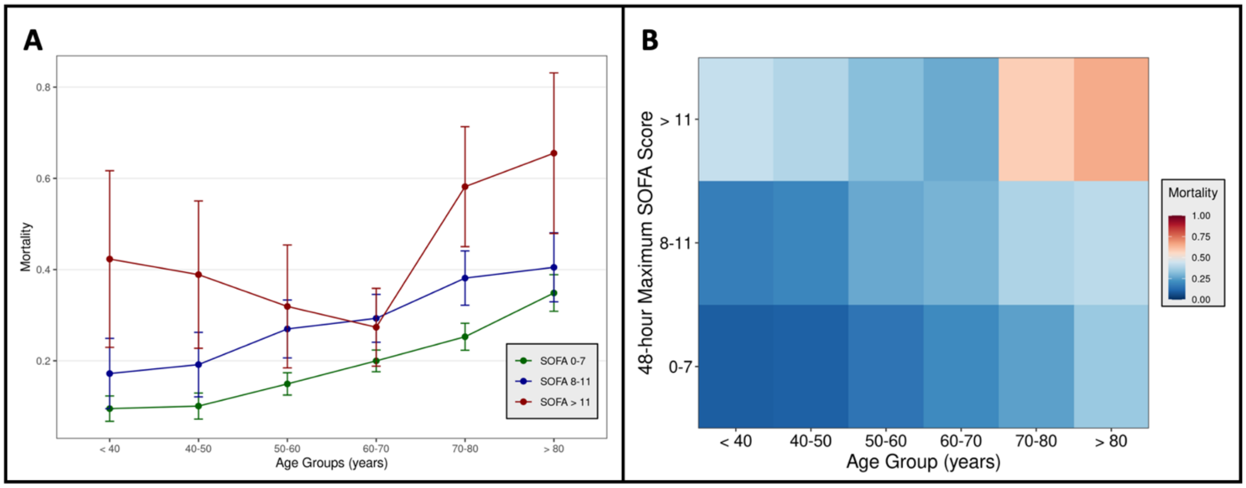

Figure S5: Association between Mortality and Age in the Derivation Cohort. Panel A shows observed mortality for various age groups, stratified by 48-hour maximum SOFA score at the beginning of each LSE, for the training dataset of $N = 5,545$ LSEs. The SOFA score stratification was done using the New York State Ventilator Allocation Guideline groups (SOFA score 0 – 7, 8 – 11, and > 11). Error bars show the 95% confidence interval calculated using the standard equation for a proportion. Panel B shows observed mortality at various age and SOFA score groups. LSE = life support episode, SOFA = Sequential Organ Failure Assessment

Figure S6. Association between Mortality and Age In the Validation Cohort.

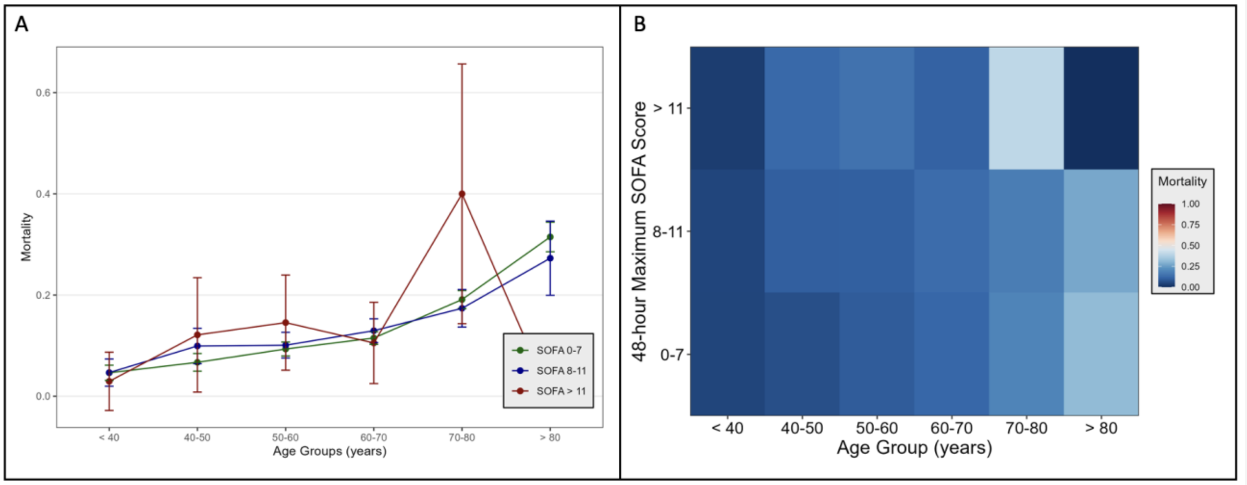

Figure S6, Panel A shows observed mortality for various age groups, stratified by 48-hour maximum SOFA score at the beginning of each LSE, for the validation cohort of *N* = 11,166 LSEs. The SOFA score stratification was done using the New York State Ventilator Allocation Guideline groups (SOFA score 0 – 7, 8 – 11, and > 11). Error bars show the 95% confidence interval calculated using the standard equation for a proportion. Panel B shows observed mortality at various age and SOFA score groups for the validation data. LSE = life support episode, SOFA = Sequential Organ Failure Assessment

Figure S7. Life Support Episode Mortality by Deciles of SOFA+Age Score.

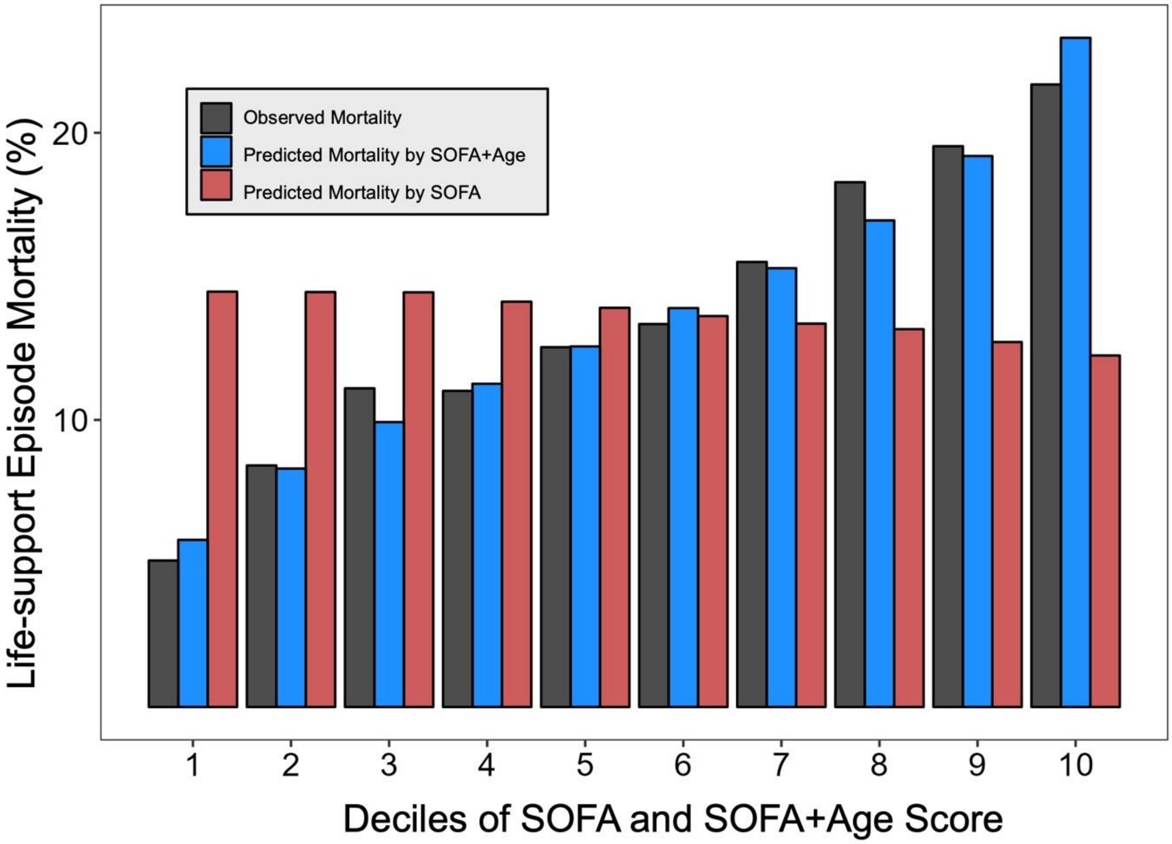

Figure S7 shows observed and predicted LSE mortality at each decile of the SOFA+Age score or SOFA score. Both the SOFA+Age score and SOFA score were recalibrated to predict LSE mortality in the validation set. The Hosmer-Lemeshow statistic for predicted mortality by SOFA+Age score versus observed mortality was 7.331, $p = 0.50$. The Hosmer-Lemeshow statistic for predicted mortality by SOFA score versus observed mortality was 127.09, $p < 0.001$. LSE = life support episode, SOFA = Sequential Organ Failure Assessment

Figure S8. Calibration Curves for Logistic Regression using SOFA Score in Derivation and Validation Cohorts.

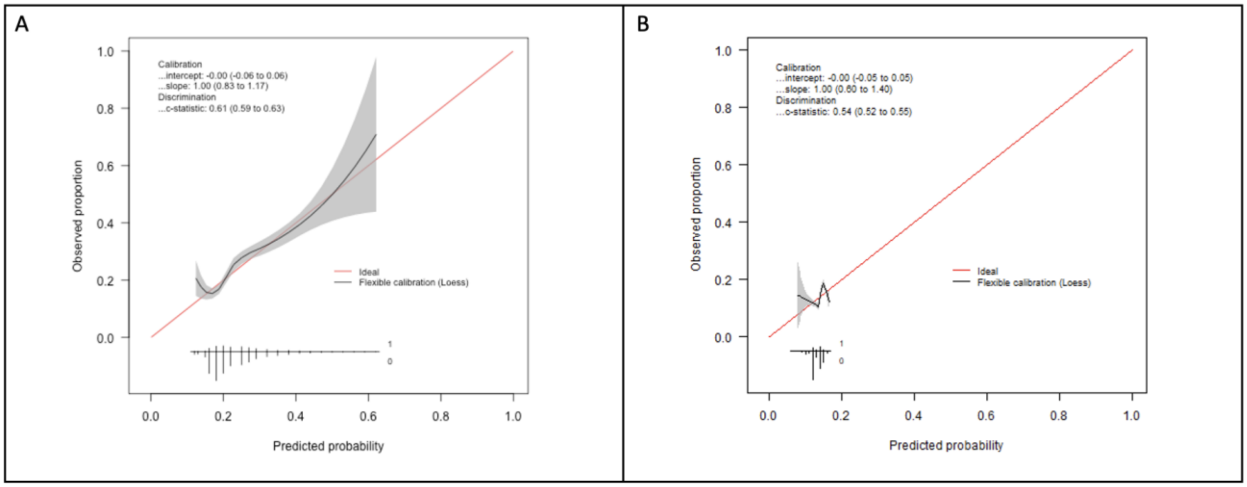

Figure S8 shows calibration curves for the logistic regression model using 48-hour maximum SOFA score at the beginning of LSE only to predict LSE mortality. Panel A shows the calibration curve for the model in the derivation cohort and panel B shows the calibration curve for the model in the validation cohort. The model was recalibrated to the validation cohort prior to making predictions. Calibration method from Van Calster, B., Nieboer, D., Vergouwe, Y., De Cock, B., Pencina, M.J., Steyerberg, E.W. (2016). A calibration hierarchy for risk models was defined: from utopia to empirical data. Journal of Clinical Epidemiology, 74, pp. 167-176. SOFA = Sequential Organ Failure Assessment, LSE = life support episode

Figure S9. Calibration Curves for Logistic Regression using SOFA Categories in Derivation and Validation Cohorts.

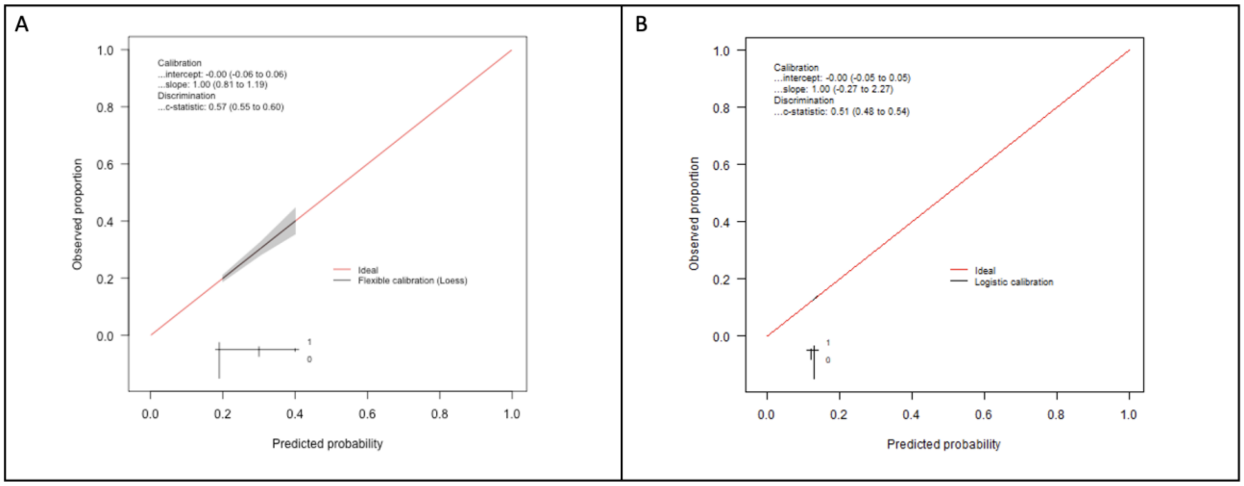

Figure S9 shows calibration curves for the logistic regression model using categories of 48-hour maximum SOFA score at the beginning of LSE to predict LSE mortality. Categories were based on New York State Ventilator Allocation Guidelines groups (SOFA 0 – 7, 8 – 11, or > 11). Panel A shows the calibration curve for the model in the derivation cohort and panel B shows the calibration curve for the model in the validation cohort. The model was recalibrated to the validation cohort prior to making predictions. Calibration method from Van Calster, B., Nieboer, D., Vergouwe, Y., De Cock, B., Pencina, M.J., Steyerberg, E.W. (2016). A calibration hierarchy for risk models was defined: from utopia to empirical data. Journal of Clinical Epidemiology, 74, pp. 167-176. SOFA = Sequential Organ Failure Assessment, LSE = life support episode

Figure S10. Calibration Curve for Logistic Regression using SOFA+Age score in Derivation and Validation Cohorts.

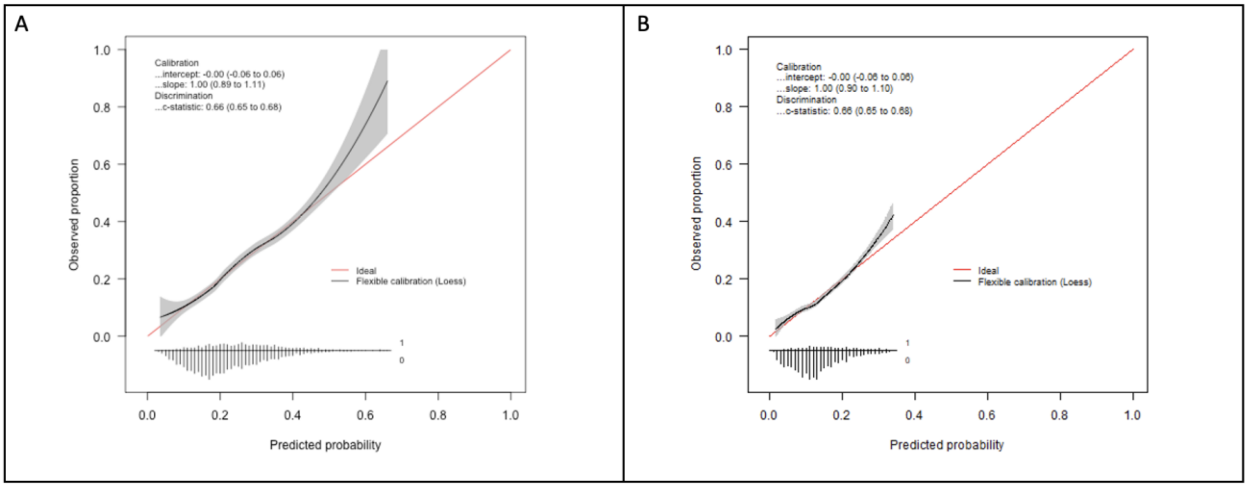

Figure S10 shows calibration curves for the logistic regression model using the SOFA+Age score to predict LSE mortality. Panel A shows the calibration curve for the model in the derivation cohort and panel B shows the calibration curve for the model in the validation cohort. The model was recalibrated to the validation cohort prior to making predictions. Calibration method from Van Calster, B., Nieboer, D., Vergouwe, Y., De Cock, B., Pencina, M.J., Steyerberg, E.W. (2016). A calibration hierarchy for risk models was defined: from utopia to empirical data. Journal of Clinical Epidemiology, 74, pp. 167-176. LSE = life support episode

Figure S11. Receiver-operator characteristic curves for predictive models using SOFA score or SOFA+Age score (first LSE only).

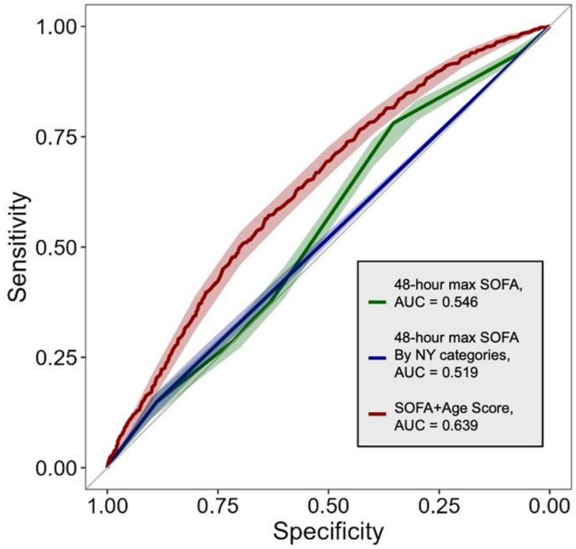

Figure S11 shows the ROC curves for three different logistic regression models fit to predict mortality at the end of an LSE in the validation cohort using only the first LSE from each patient. Two of the models were fit using a single variable: either 48-hour maximum SOFA score at the beginning of the LSE or categories of 48-hour maximum SOFA score at the beginning of LSE based on New York State Ventilator Allocation Guidelines (SOFA 0 – 7, 8 – 11, or > 11). The last model was fit using the SOFA+Age score (a novel score derived from age (in years) and 48-hour maximum SOFA score at the beginning of the LSE). The shaded region surrounding each curve shows the 95% confidence interval calculated by 2000 stratified bootstrap replicates. In the validation data, paired DeLong's test for SOFA score alone versus SOFA+Age score: $p < 0.001$. Paired DeLong's test for SOFA score categories versus SOFA+Age score: $p < 0.001$. ROC = receiver-operator characteristic, LSE = life support episode, SOFA = Sequential Organ Failure Assessment

Figure S12. Life Support Episode Mortality by Deciles of SOFA+Age or SOFA Score (first LSE only).

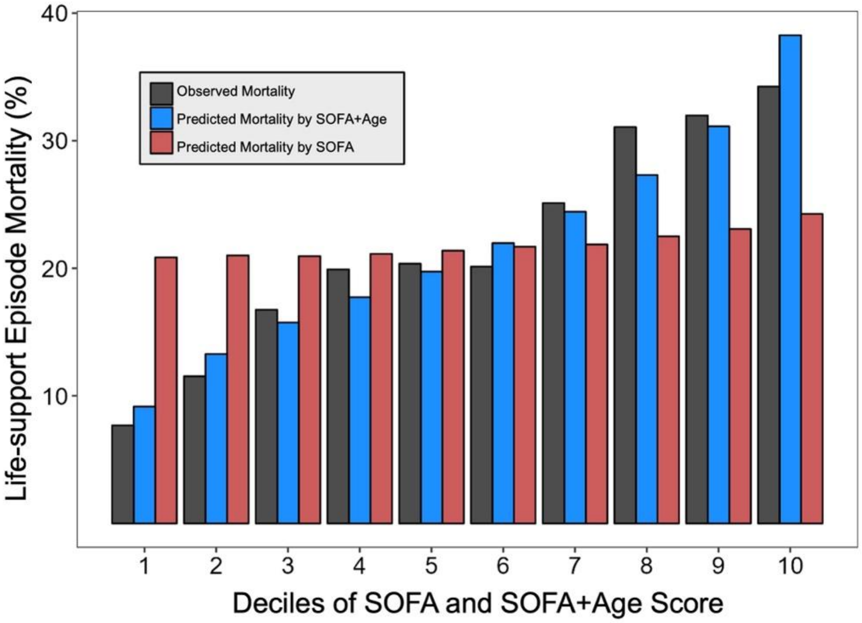

Figure S12 shows observed and predicted LSE mortality at each decile of the SOFA+Age score or SOFA score using only the first LSE for each patient. There was no significant difference between observed mortality and predicted mortality by SOFA+Age score, $p = 0.2$ by the Hosmer-Lemeshow test. There was a significant difference between observed mortality and predicted mortality by SOFA score, $p < 0.001$ by the Hosmer-Lemeshow test. LSE = life support episode, SOFA = Sequential Organ Failure Assessment

Figure S13. Life support episode mortality by age group in derivation and validation sets (first LSE only).

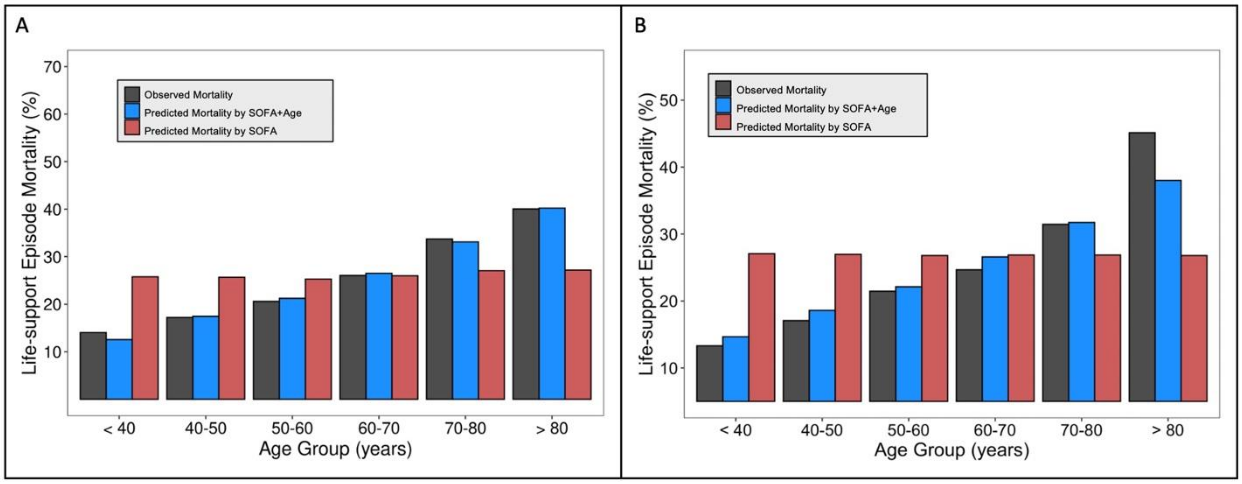

Figure S13 shows observed and predicted LSE mortality at various age groups using only the first LSE for each patient. Panel A shows data from the derivation cohort and Panel B shows data from the validation cohort. Both the SOFA+Age score and SOFA score were recalibrated to the validation data prior to making predictions in the validation data. LSE = life support episode, SOFA = Sequential Organ Failure Assessment

Figure S14. Life support episode mortality by racial-ethnic groups (first LSE only).

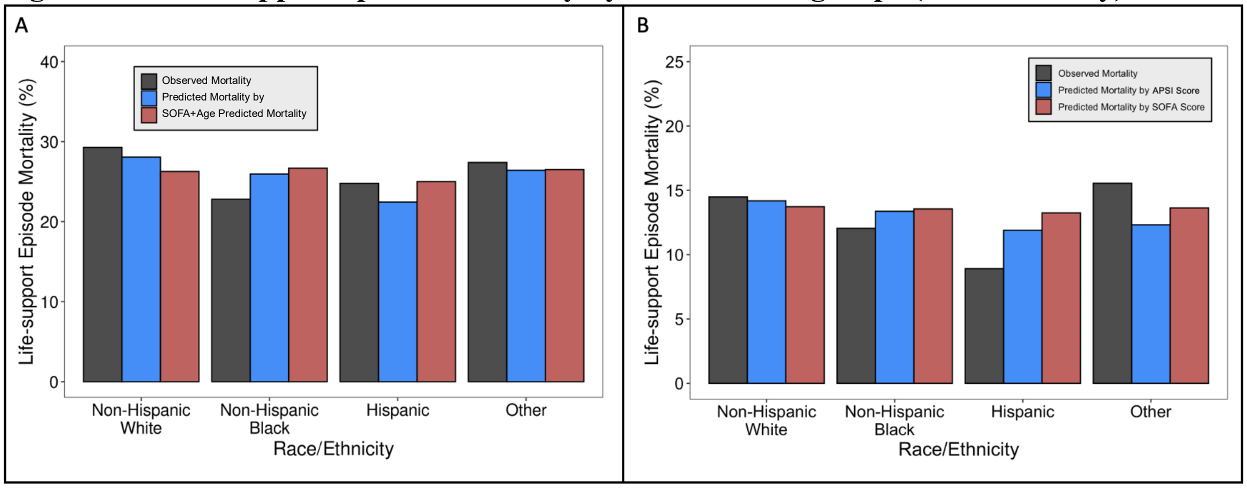

Figure S14 shows observed and predicted LSE mortality for different racial-ethnic groups using only the first LSE for each patient. Panel A shows data from the derivation set and panel B shows data from the validation set. LSE = life support episode, SOFA = Sequential Organ Failure Assessment

Figure S15. HHS OCR Statement on Civil Rights and COVID-19.

NON-DISCRIMINATION IN CRISIS STANDARDS OF CARE

At the beginning of the COVID-19 public health emergency, OCR made clear that civil rights laws are not suspended or waived in times of disaster, including COVID-19 - PDF. As set forth in this March 2020 bulletin: "OCR enforces Section 1557 of the Affordable Care Act and Section 504 of the Rehabilitation Act which prohibit discrimination on the basis of disability in HHS funded health programs or activities. These laws, like other civil rights statutes OCR enforces, remain in effect. As such, persons with disabilities should not be denied medical care on the basis of stereotypes, assessments of quality of life, or judgments about a person's relative "worth" based on the presence or absence of disabilities or age. Decisions by covered entities concerning whether an individual is a candidate for treatment should be based on an individualized assessment of the patient based on the best available objective medical evidence."

As a result of complaints filed with OCR and requests for technical assistance, OCR has worked with states and within HHS to address non-discrimination in crisis standard of care plans and practices.

Examples of how OCR has worked with states, HHS components, and the Health Care Resilience Crisis Standards of Care Taskforce to operationalize these principles appear below, along with conference presentations and podcasts on this topic in which OCR has participated.

DISABILITY AND CRISIS STANDARDS OF CARE

Best Practices from OCR's Work with States and Other Entities on Crisis Standards of Care

- Resource allocation decisions should be based on individualized assessment of each patient using best available objective medical evidence concerning likelihood of death prior to or imminently after hospital discharge
- Such assessments should not use categorical exclusion criteria on the basis of disability or age; judgments as to long-term life expectancy; evaluations of the relative worth of life, including through quality of life judgments, and should not deprioritize persons on the basis of disability or age because they may consume more treatment resources or require auxiliary aids or supports.
- When using prognostic scoring systems with patients with underlying disabilities, reasonable modifications may be necessary for accurate use.
- Healthcare providers should not "steer" patients into agreeing to the withdrawal or withholding of life-sustaining treatment or require patients or their families to consent to a particular advanced care planning decision in order to continue to receive services from a facility. Patients must be given information on the full scope of available alternatives.
- Providers should not consider for re-allocation a ventilator or other piece of life-sustaining equipment that is brought to the hospital by a patient whose life is dependent on that equipment

AGE AND CRISIS STANDARDS OF CARE

On January 14, 2021, OCR announced it worked collaboratively with the State of NC, the North TX Mass Critical Service to revise each entity's crisis standards of care ("CSC") guidelines to reflect best practices for serving individuals with disabilities and the elderly. After OCR provided technical assistance to each entity through a collaborative process, they issued CSC plans that incorporated the following provisions:

- Prohibition on the use of a patient's long-term life expectancy as a factor in the allocation and re-allocation of scarce medical resources;
- Prohibition on the use of categorical exclusion criteria, instead requiring an individualized assessment based on the best available objective medical evidence;
- Prohibition on the use of resource-intensity and duration of need as criteria for the allocation or re-allocation of scarce medical resources. This protects patients who require additional treatment resources due to their age or disability from being given a lower priority to receive life-saving care due to such need;
- Inclusion of language stating that reasonable modifications to the use of clinical instruments for assessing likelihood of short-term survival should be made when necessary for accurate use with patients with underlying disabilities.
- Inclusion of new protections against providers "steering" patients into agreeing to the withdrawal or withholding of life-sustaining treatment, clarifying that patients may not be subject to pressure to make particular advanced care planning decisions, must be given information on the full scope of available alternatives, and that providers may not impose blanket "Do Not Resuscitate" policies for reasons of resource constraint, or require patients to consent to a particular advanced care planning decision in order to continue to receive services from a facility; and
- Inclusion of language stating that hospitals should not re-allocate personal ventilators brought by a patient to an acute care facility to continue pre-existing personal use with respect to a disability. Under this language, long-term ventilator users will be protected from having a ventilator they take with them into a hospital setting taken from them to be given to someone else.

In addition to the agency's work with covered entities regarding CSC guidelines, OCR recently worked collaboratively with the National Academy of Medicine (NAM) to advise on the development of a statement on CSC guidelines during COVID-19, issued by the NAM and nine other national organizations reflecting key best practices for CSC plans.

In the December 18, 2020 National Organizations Call for Action to Implement Crisis Standards of Care During COVID-19 Surge, several recommendations address resource allocation decisions based on age. In particular, the recommendations state:

- However, such assessments should NOT use ***categorical exclusion criteria*** on the basis of disability or age; judgments as to long-term life expectancy; evaluations of the relative worth of life, including through quality of life judgments, and should NOT deprioritize persons on the basis of disability or age because they may consume more treatment resources or require auxiliary aids or supports."

OCR is the statutorily designated federal agency responsible for coordination of all civil rights regulations promulgated by federal agencies under the Age Discrimination Act. Under OCR's regulations, explicit mention of age as a basis (even if one factor among many) for a denial or de-prioritization with respect to federally funded benefits is generally prohibited. 45 CFR 91.11. However, methods for resource allocation that may have a disproportionate negative impact correlated to age can be used if "the factor bears a direct and substantial relationship to the normal operation of the program or to the achievement of a statutory objective." 45 CFR 91.14. Under 45 CFR 91.13 of OCR's regulations, age can be explicitly considered if:

(a) Age is used as a measure or approximation of one or more other characteristics; and
(b) The other characteristic(s) must be measured or approximated in order for the normal operation of the program or activity to continue, or to achieve any statutory objective of the program or activity; and
(c) The other characteristic(s) can be reasonably measured or approximated by the use of age; and
(d) The other characteristic(s) are impractical to measure directly on an individual basis.
Under 45 CFR 91.15, the recipient of federal funds has the burden of proving that use of age falls within the exceptions. This means statements saying providers may use age distinctions are generally not appropriate because each provider has to justify such use individually.

Figure S15 shows screenshots from a web page titled "Civil Rights and COVID-19" from the HHS and OCR. URL: https://www.hhs.gov/civil-rights/for-providers/civil-rights-covid19/index.html. Accessed 2-24-23.
